# Effects of summer savory (*Satureja hortensis* L.) and sweet corn (*Zea mays* L. saccharata) intercropping on crop production and essential oil profiles of summer savory

**DOI:** 10.7717/peerj.14753

**Published:** 2023-01-30

**Authors:** Ruhollah Naderi, Farzad Bijani, Akbar Karami, Bhagirath S. Chauhan, Todd P. Egan

**Affiliations:** 1 School of Agriculture, Shiraz University, Shiraz, Iran; 2 The University of Queensland, Queensland, Australia; 3 Elmira College, New York, United States

**Keywords:** Sustainable agriculture, Medicinal plants, Industrial crops, Land equivalent ratio

## Abstract

A 2-year field experiment evaluated the effects of sweet corn-summer savory intercropping on crop productivity and essential oil (EO) composition of summer savory. Five cropping patterns of Corn 100%:Savory 0%, C75:S25, C50:S50, C25:S75, and C0:S100 were tested. The highest corn yield (2,440 kg ha^−1^) was obtained in a corn monoculture, but was not significantly different from C75:S25 or C50:S50. However, in both years the highest savory yield was obtained in S100 (793.3 g m^−2^ and 816.6 g m^−2^, respectively). Savory yields decreased as the proportion of corn increased. The land equivalent ratios in C25:S75, C50:S50, and C75:S25 were 1.54 ± 0.07, 1.56 ± 0.03, and 1.35 ± 0.1, respectively. Monocropped savory had the highest EO value followed by C25:S75 and C50:C50. However, no significant differences were found among these three treatments. Gas chromatography-mass spectrometry (GC–MS) analysis showed that the major components were carvacrol (35.88%–42.96%), γ-terpinene (18.45%–20.03%), ρ-cymene (11.77%–12.24%), and α-terpinene (2.75%–3.96%). The highest amount of carvacrol was recorded in C25:S75 (42.96%). This study suggests that intercropping of corn and savory represents an effective sustainable strategy, especially for smallholders, as a way to increase their overall land productivity and to improve the quality of savory’s EO.

## Introduction

In recent years the global demand for both processed and fresh sweet corn (*Zea mays* L. saccharata) from the Poaceae family, has greatly increased (Williams, 2014), and this has led to an approximate one million hectare increase in its cultivation throughout the globe (Tang et al., 2017). In the Iranian Province of Fars, water shortage is a major problem. However, this has not prevented an increase in sweet corn production due to its increased market price. A contributing factor to sweet corn’s rise in popularity may be due to the many ways it can be enjoyed (Alijani, Bahrani & Kazemeini, 2019). Fresh kernels can be consumed from marketable ears, fed to livestock as forage, and baby corn can be processed. Sweet corn for human consumption is harvested when kernel development is at the ‘milk’ stage, which is earlier than for field corn. Harvesting corn early reduces water and nitrogen consumption, and eliminates the delay before sowing the subsequent crop (Alijani, Bahrani & Kazemeini, 2019). Sweet corn’s shorter growing season is an additional incentive for crop producers (Teasdale, Abdul-Baki & Park, 2008).

Summer savory (*Satureja hortensis* L.) from the Lamiaceae family, is a medicinal and spice plant distributed mostly in the Mediterranean (Bakhshian et al., 2022). Iran’s flora supports fourteen *Satureja* species, eight of which are endemic to Iran (Taban, Saharkhiz & Hadian, 2013). This plant is used in both the food (Wójcik-Stopczyńska, Jakubowska & Reichelt, 2009; Michalczyk & Banaś, 2014) and pharmaceutical industries (Kapka-Skrzypczak & Król, 2011; Adaszyńska & Swarcewicz, 2012).

Intercropping is the cultivation of two or more crops concurrently in the same location, and is predominantly employed to enhance crop productivity per unit area (Brooker et al., 2015; Olasantan, Ezumah & Lucas, 1996). Intercropping remarkably enhances crop yields while diminishing agriculture’s environmental footprint, and as a result, this technique benefits both the environment and the economy (Tilman, 2020).

Farmers in developing countries often employ strategies such as intercropping to enhance total land productivity (Mohammed et al., 2022). Intercropping systems may combine food crops with medicinal plants to increase per unit yields, as well as to improve medicinal plant quality. For instance, Fallah et al. (2018) found that dragonhead produced the most of geranyl acetate (*Dracocephalum moldavica* L.) F. Lamiaceae when intercropped with soybean (*Glycine max* L.). Amani Machiani et al. (2018) found that intercropping peppermint (*Mentha piperita* L.) with faba bean (*Vicia faba* L.) increased menthol content, but decreased menthofuran and pulegone contents in peppermint.

Presently, sweet corn and summer savory are monocrops in southern Iran. However, because of the industrial importance of sweet corn and the medicinal importance of summer savory, agronomists and farmers have recently begun to investigate intercropping and other methods to enhance the yield and quality of these crops. A literature search indicates that the compatibility of sweet corn and summer savory in an intercropping system has yet to be investigated.

Therefore, the objectives of this study were: (i) to determine the benefits of summer savory-sweet corn intercropping over monocropping, and (ii) to evaluate the yield and chemical composition of summer savory essential oils (EOs) from intercropped systems.

## Materials and Methods

### Field site description

A 2-year field study began in 2019 at the Research Field of Shiraz University, School of Agriculture, Iran (29.73693°N, 52.58225°E; 1,810 m above sea level). The soil was a silty loam (fine, mixed mesic, Typic Calcixerpets) with a pH of 8, EC of 0.65 dS m^−1^, total N 0.06%, available P of 50 mg kg^−1^, available K of 100 mg kg^−1^, and 0.7% organic matter.

### Plant material and cultivation

This experiment was conducted as a randomized complete block design with three replicates. Treatments included five cropping patterns of sweet corn and savory seeds (monocropped sweet corn (C), monocropped savory (S), and three intercropping ratios of sweet corn and savory as 75% sweet corn, 25% savory (C75:S25), 50% sweet corn, 50% savory (C50:S50), and 25% sweet corn, 75% savory (C25:S75). Sweet corn seeds were obtained from the Seed and Plant Improvement Institute (Karaj, Iran), and savory seeds were obtained from the Pakan Bazar Company, Isfahan, Iran.

Seedbeds were prepared using a mould-board plow, disk harrow, and leveler, respectively, in a field that was uncultivated the previous growing season. Maize caryopses and savory seeds were planted on 14 May 2019 and 24 May 2021. Cultivation was accomplished as per Naderi et al. (2022) such that between-row and within-row spacing was 75 and 17 cm, respectively, for sweet corn. Summer savory seeds were broadcast into the furrows of intercropped treatments. In monoculture plantings, savory seeds were broadcast into the furrows as well as on the ridge tops. Each plot was 3 m long and consisted of four rows. Maize monoculture density was 16 plants m^−2^ and savory was 40 plants m^−2^. Intercropped plots were proportions based on these monocrop densities. Therefore, corn to savory of C75:S25 had 12 corn seeds:30 savory seeds, C50:S50 had C8:S20, and C25:S75 had C4:S10. Seeds were irrigated upon planting, twice every 6 days, then as needed. Weeds were removed manually and regularly, so no chemical herbicides were needed.

### Harvesting of sweet corn and savory

To determine sweet corn and savory yield a 1 m^−2^ section from the middle two rows of each plot was harvested on 27 August in 2019 and 24 August in 2021, respectively. Maize ear fresh weight was determined. Savory shoot tissue was harvested, dried in the shade at room temperature for 2 weeks, dry weight was determined, then stored for essential oil determination.

### Essential oil isolation

The British Pharmacopoeia (BPC, 1998) method was used to extract essential oils (EO) from 30 g of ground, dried plant material which was hydrodistilled for 3 h using a Clevenger type apparatus (IROST, Tehran, Iran). Essential oil samples were dehydrated with anhydrous Na_2_SO_4_ and dark refrigerated (4 °C) in glassware to await analysis.

### Analysis and identification of essential oils compounds

An Agilent (Santa Clara, CA, USA) gas chromatograph series 7890-A with a flame ionization detector (FID) was used to perform GC analysis. Essential oils were isolated using an HP-5 fused silica capillary column (30 m × 0.32 mm i.d.; film thickness 0.25 μm). The injector temperature was maintained at 250 °C, and the detector at 280 °C. The mobile phase was nitrogen gas flowing at 1 mL/min. Oven temperature was initially 60–210 °C at a rate of 4 °C/min, then 240 °C at a rate of 20 °C/min, and finally held isothermally for 8.5 min. The split ratio was 1:50.

An Agilent gas chromatograph with an HP-5MS fused silica capillary column (30 m × 0.25 mm i.d.; film thickness 0.25 μm) coupled with an 5975-C mass spectrometer was used to perform GC-MS analysis. The mobile phase was helium with an ionization voltage of 70 eV. The ion source was 230 °C, with an interface temperature of 280 °C. The mass ranged from 45 to 550 amu, and oven temperatures were as above. Retention index values at determined temperatures for n-alkanes (C8-C25), and essential oils on an HP-5 column under the above chromatographic conditions were used to identify essential oil constituents. Individual essential oils were determined by comparing each mass spectrum with those of a mass spectra library, or with authentic compounds. Confirmation was accomplished by comparing retention indices with authentic compounds (Alkane standard solution n-alkanes (C_8_-C_25_) or with those reported in the literature (Adams, 2007). Relative area percentages obtained *via* FID did not employ correction factors for quantification.

### Land equivalent ratio

Land Equivalent Ratio (LER) is defined as the land area needed by monocrops to produce the same yield produced by intercrops (Willey, 1979). A value of LER >1.0 shows a yield advantage of intercropping over monocropping, and vice versa. LER was calculated using the following formula (Ofori & Stern, 1987):

$LER = \displaystyle{{Yci} \over {Ycm}} + \displaystyle{{Ysi} \over {Ysm}}$

where Yci and Ysi are the yields of sweet corn and savory in the intercropping system, respectively, and Ycm and Ysm are the yields of sweet corn and savory in sole cropping.

### Statistical analysis

Statistically significant differences among means were determined *via* an analysis of variance (ANOVA). When a significant difference was found, individual treatment means were compared using the Duncan’s multiple range test (*P* < 0.05). Since there were no significant (*P* > 0.05) interactions between the 2019 and 2021 growing seasons for cropping pattern of sweet corn yield, essential oil production, and LER, the data from both years were combined for these parameters. However, there was a significant (*P* < 0.05) difference between the 2019 and 2021 growing seasons for the savory cropping pattern, so each year’s data were reported separately for this parameter (SAS version 9.1, 2009; SAS Institute, Cary, NC, USA).

## Results and discussion

### The effects of cropping patterns on sweet corn yield

**Figure 1:**
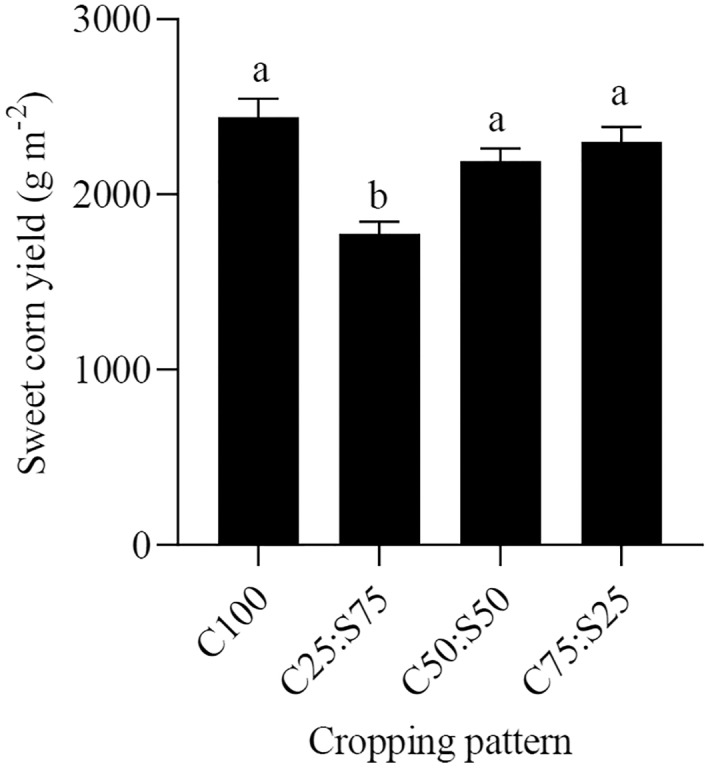
Corn yield as affected by cropping pattern; C100, C25:S75, C50:S50, C75:S255 refer to a corn monoculture and intercropping ratios and corn: summer savory (of plant densities used in respective monocultures). Data were pooled across both years. Different letters between bars denote statistically significant differences (*P* < 0.05).

Cropping pattern had a significant effect on sweet corn yield (F = 64.35, *P* < 0.05). The highest sweet corn yield (2,440 kg ha^–1^kg ha^–1^) was obtained in the sweet corn monoculture, but it was not significantly different from C75:S25 and C50:S50 (Fig. 1). This indicates that sweet corn-summer savory intercropping is superior to monocropping in terms of yield output. Higher yields gained in intercropped fields utilize resources in an integrative manner, as they do not compete for the same available resources including water, nutrients, and light within a niche (Lithourgidis et al., 2011). Selecting a suitable planting ratio and density in intercropping systems can enhance the economic yield of these systems due to increased root biomass, as well as the optimal exploitation of environmental conditions (Raza et al., 2019). Previous studies have found that intercropping medicinal plants with field crops such as dragonhead (*Dracocephalum moldavica*) (Fallah et al., 2018) and fennel (*Foeniculum vulgare* L.) (Rezaei-Chiyaneh et al., 2020) were superior to their monocropping with respect to yield output.

### The effects of cropping patterns on savory biomass yield

**Figure 2:**
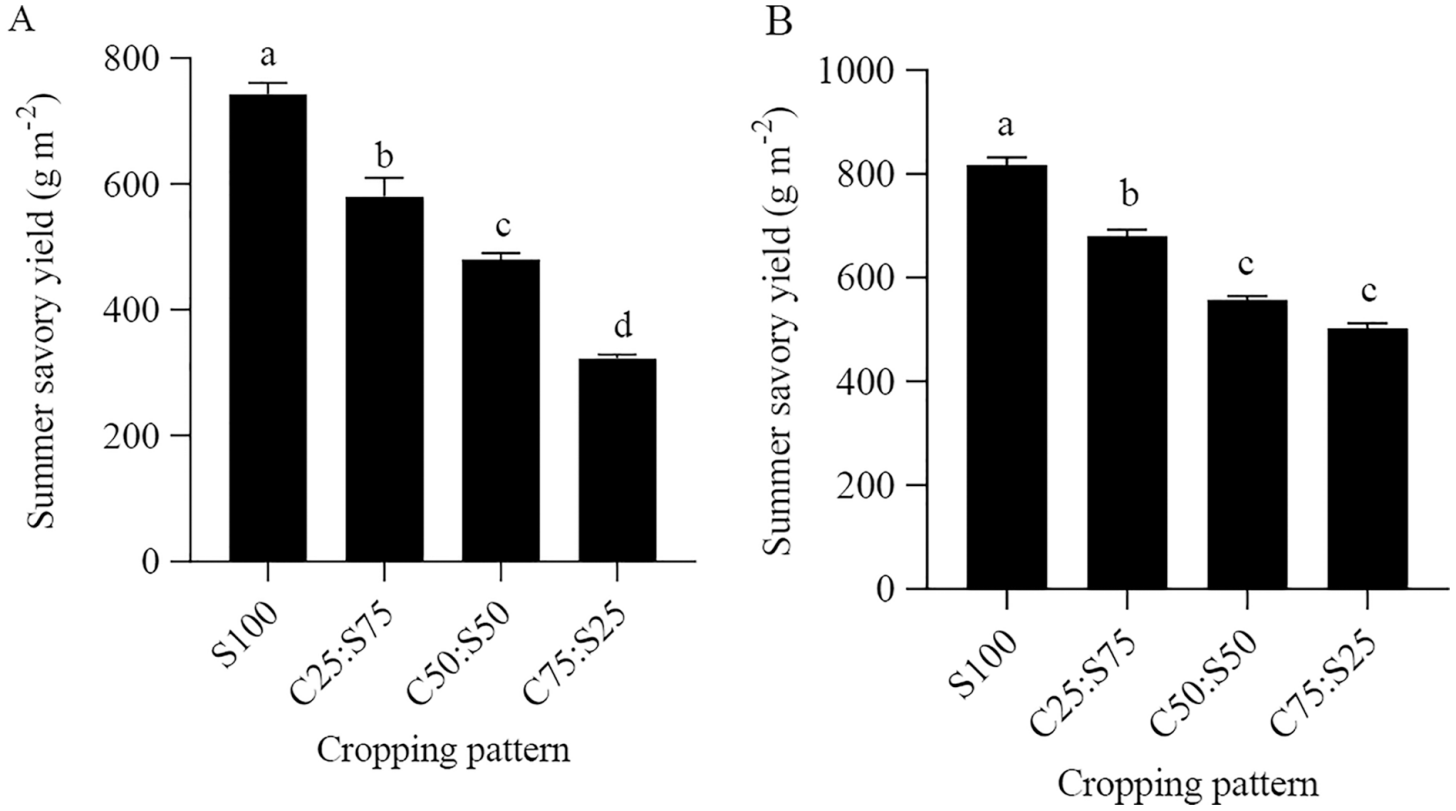
Effects of cropping pattern on savory yield in 2019 (A) and 2021 (B). S100, C25:S75, C50:S50, and C75:S25 refer to summer savory alone and intercropping ratios for sweet corn: summer savory (of plant densities used in respective monocultures). Different letters between bars denote statistically significant differences (*P* < 0.05).

Summer savory yield was significantly affected by cropping pattern in 2019 and 2021 (F = 116.11, *P* < 0.05; F = 92.07, *P* < 0.05). In both years, the highest savory yield was obtained in S100 (793.3 and 816.6 g m^−2^, respectively). Summer savory yields decreased as the proportion of sweet corn increased in the mixture (Figs. 2A and 2B). This is highly likely because of the differences in sowing densities used in cropping patterns; thus, the sowing density for monocropping systems was higher than the intercropping systems. Our results are in accordance with those of Fallah et al. (2018) who found that in an intercropping of soybean and dragonhead, the highest yield of dragonhead was achieved in its monoculture.

### The effects of cropping pattern on land equivalent ratio

Cropping pattern significantly affected LER (F = 3.7, *P* < 0.05). The land equivalent ratio in all mixtures exceeded the unitary value of 1.0, indicating a yield advantage of intercropping over monocropping, which is because of more efficient land utilization as well as better use of available plant resources (Banik et al., 2000). The land equivalent ratio in C25:S75, C50:S50, and C75:S25 were 1.54 ± 0.07, 1.56 ± 0.03, and 1.35 ± 0.1, respectively, showing that in monocropping systems, 54%, 56%, and 35% more land area is needed to gain an equal yield to that of the intercropping system. This also noted that land-use efficiency in intercropping is greater than monocropping (Midya et al., 2005; Agegnehu, Ghizaw & Sinebo, 2006). Achieving higher yields in intercropping systems means that less land is required per unit of food production, thus these systems would potentially diminish future land clearing and greenhouse gas emissions (Tilman, 2020).

Similarly, increased LER above 1.0 has been reported in dragonhead and soybean (Fallah et al., 2018), wheat *(Triticum aestivum*) and bean *(Vicia faba)* (Cannon, Kamalongo & Conway, 2020), fennel and common bean (Rezaei-Chiyaneh et al., 2020), and saffron and pumpkin/watermelon (Koocheki, Rezvani Moghaddam & Seyyedi, 2019) intercroppings. These examples highlight the advantages of intercropping compared to monocropping, potentially due to utilizing environmental resources more efficiently.

### The effects of cropping pattern on essential oil content of summer savory

**Figure 3:**
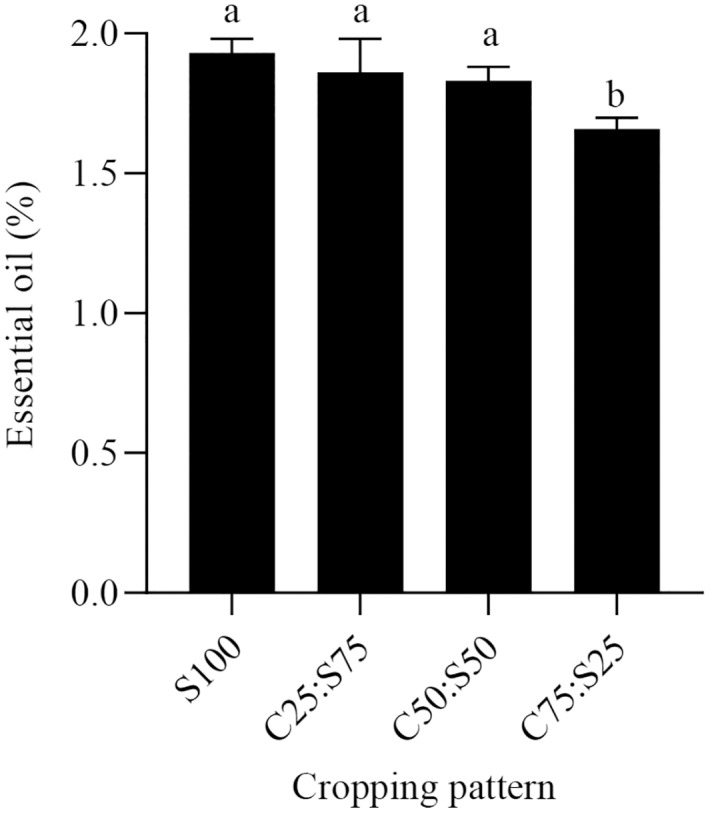
Effects of cropping pattern on essential oil in savory. S100, C25:S75, C50:S50, and C75:S25 refer to summer savory alone, intercropping ratio for sweet corn: summer savory (of plant densities used in respective monocultures). Data were pooled across both years. Different letters between bars denote statistically significant differences (*P* < 0.05).

The essential oil content of summer savory was affected by cropping pattern (F = 5.11, *P* < 0.05). Monocropped summer savory had the highest EO value, following by C25:S75 and C50:C50. However, no significant differences were found among these three treatments. The C75:S25 treatment had significantly lower amounts of EO (Fig. 3).

Similar to the present findings, Rezaei-Chiyaneh et al. (2020) noted that the EO content of fennel (*Foeniculum vulgare* L.) increased in a common bean and fennel intercropping. Weisany, Raei & Ghassemi-Golezani (2016) also reported that the EO content of dill increased in a common bean and dill intercropped system. In contrast, Fallah et al. (2018) found no significant effect of intercropping on EO content in dragonhead.

Production of secondary metabolites such as essential oil is associated with higher rates of photosynthetic activity. Thus, the availability of nutrients, specifically nitrogen and phosphorous, that enhances the level of photosynthetic activity could lead to greater production of essential oil in medicinal plants (Croteau, Burbott & Loomis, 1972). Nitrogen plays a crucial role in the biosynthesis of several organic compounds, such as amino acids and enzymes, in which these compounds also play a pivotal role in the biosynthesis of essential oil constituents (Koeduka et al., 2006). Our EO results may indicate that sweet corn in C25:S75 and C50:S50 did not compete for soil nutrients with summer savory, and thus did not reduce the available nutrients and eventually did not decrease the amount of essential oil in summer savory. These results coupled with summer savory and sweet corn yields would enable farmers to prioritize the intercropping pattern based on target crop species. For instance, if the target crop is sweet corn, then an intercropping pattern of C75:S25 will significantly add to the yield of sweet corn, however, the yields and EO of summer savory would be sub-optimal.

### The effects of cropping pattern on summer savory essential oil composition

**Figure 4:**
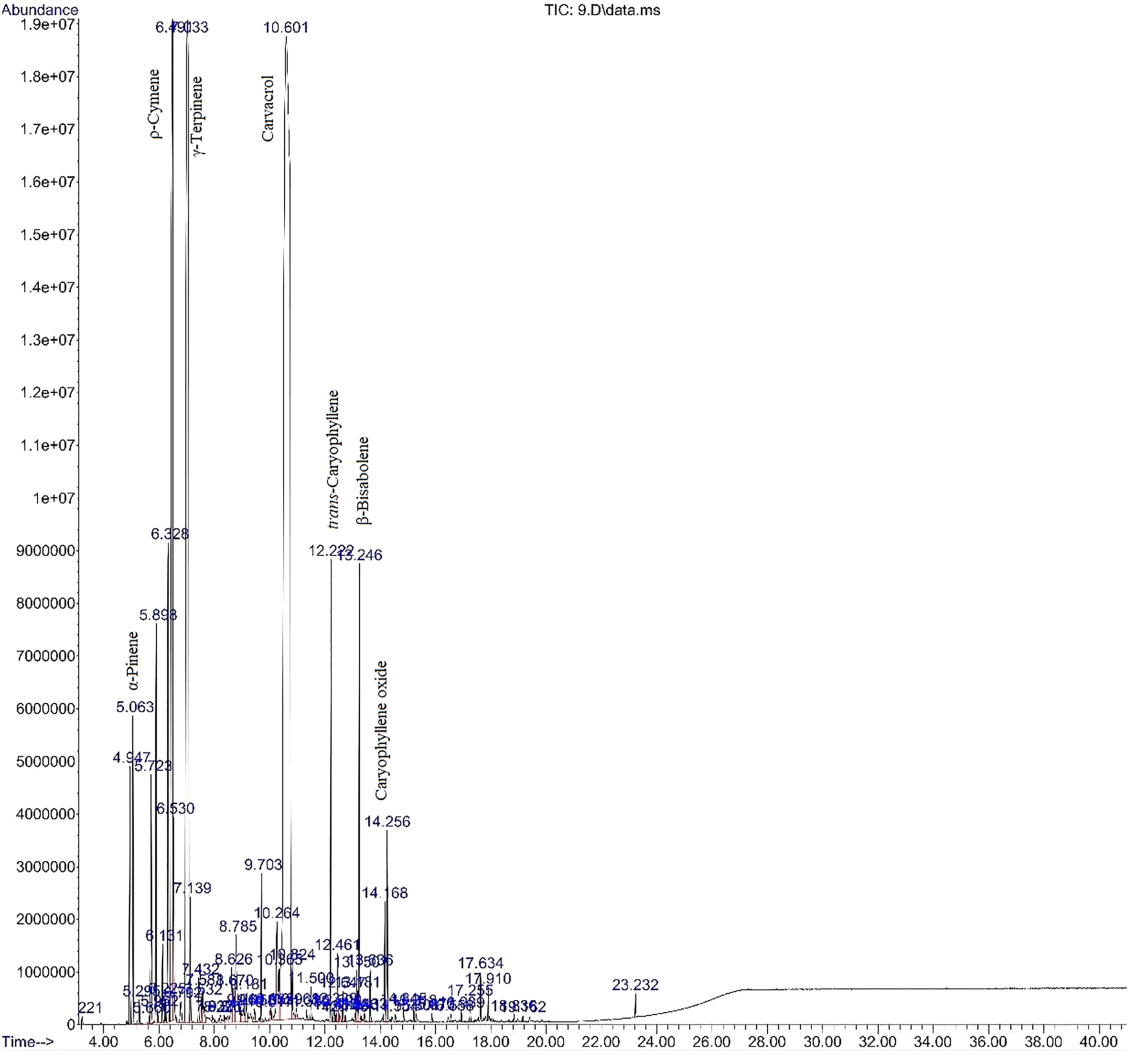
GC/MS chromatogram of summer savory sample with major compounds peak identification.

Summer savory EO composition was analyzed *via* GC–MS (Fig. 4), and 43 compounds were identified (Table 1). The major components were carvacrol (35.88%–42.96%), γ-terpinene (18.45%–20.03%), ρ-cymene (11.77%–12.24%), and α-terpinene (2.75%–3.96%). Present results concur with those obtained by Taban, Saharkhiz & Naderi (2020), who reported that carvacrol (52.55%), γ-terpinene (30.21%), and α-terpinene (42.96%) were major components of EO of summer savory. It has been reported that variation in the essential oil components of medicinal plants could be associated with geographic adaptation, specific habitats, ecological factors, agronomic strategy, and genetic variation (Argyropoulou et al., 2007; Chauhan et al., 2016). The highest amount of carvacrol was recorded in C25:S75 (39.48%) (Table 1). Carvacrol is known as the main compound responsible for weed suppression, and it has been reported that carvacrol has a much stronger biological activity compared with ρ-cymene and γ-terpinene (Zhou et al., 2021). Although in the present study all plots were regularly hand-weeded, it was observed that in intercropped plots the presence of weeds was consistently lower than in monocropped plots. Thus, employing summer savory in intercropping may be beneficial to suppress weeds, but its potential inhibitory impacts on main crops should be further investigated. Carvacrol also has a wide range of bioactivities presumably helpful for clinical applications including antioxidant, antimicrobial, and anticancer properties (Sharifi-Rad et al., 2018). Therefore, employing summer savory in intercropping which results in increasing carvacrol is advantageous with regard to its higher quality in clinical application.

**Table 1:** Chemical components (%) in the essential oil of savory under different intercropping patterns.

No.	Compounds	RI	Relative percentages of compounds (%)			
			S100	C25:S75	C50:S50	C75:S25
1	Hexanal	801	0.032 ± 0.001	0.043 ± 0.001	0.039 ± 0.004	0.061 ± 0.01
2	α-Pinene	924	0.053 ± 0.003	–	–	–
3	α-Thujene	930	2.152 ± 0.30	1.275 ± 0.66	1.764 ± 0.64	1.087 ± 0.38
5	Camphene	954	0.196 ± 0.05	0.117 ± 0.05	0.164 ± 0.02	0.344 ± 0.02
6	Sabinene	969	0.042 ± 0.002	0.032 ± 0.006	0.043 ± 0.001	0.043 ± 0.001
7	β-Pinene	974	2.474 ± 0.27	1.501 ± 0.63	2.07 ± 0.50	1.147 ± 0.60
8	β-Myrcene	988	2.516 ± 0.19	2.094 ± 0.30	2.408 ± 0.23	1.894 ± 0.47
9	Octanol	995	0.065 ± 0.004	0.071 ± 0.001	0.074 ± 0.001	0.063 ± 0.004
10	α-Phellandrene	1,002	0.634 ± 0.04	0.413 ± 0.08	0.508 ± 0.06	0.368 ± 0.10
11	δ-3-Carene	1,008	0.175 ± 0.01	0.137 ± 0.02	0.163 ± 0.02	0.123 ± 0.04
12	α-Terpinene	1,014	3.969 ± 0.25	3.232 ± 0.35	3.768 ± 0.17	2.752 ± 0.20
13	ρ-Cymene	1,020	12.249 ± 0.28	11.777 ± 0.08	12.073 ± 0.51	12.202 ± 0.19
14	D-Limonene	1,024	1.158 ± 0.05	1.013 ± 0.07	1.118 ± 0.03	0.935 ± 0.10
15	β-ocimene	1,032	0.239 ± 0.02	0.225 ± 0.01	0.243 ± 0.01	0.026 ± 0.10
16	γ-Terpinene	1,054	20.037 ± 0.32	19.31 ± 0.03	19.6 ± 0.02	18.454 ± 0.60
17	α-Terpinolene	1,086	0.206 ± 0.006	0.223 ± 0.001	0.21 ± 0.02	0.185 ± 0.023
18	β-Terpineol	1,130	–	0.459 ± 0.01	0.519 ± 0.02	–
19	*Cis*-Sabinene Hydrate	1,098	0.764 ± 0.10	0.362 ± 0.20	0.396 ± 0.15	1.072 ± 0.15
20	*trans*-Pinocarveol	1,135	–	0.066 ± 0.01	–	0.073 ± 0.02
21	Borneol	1,169	0.442 ± 0.03	0.801 ± 0.10	0.895 ± 0.20	0.894 ± 0.06
22	Terpinene-4-ol	1,174	0.826 ± 0.06	1.03 ± 0.12	0.89 ± 0.03	0.979 ± 0.07
23	ρ-Cymen-3-ol	1,179	0.405 ± 0.05	0.455 ± 0.15	0.888 ± 0.13	1.022 ± 0.12
24	α-Terpineol	1,188	0.307 ± 0.12	0.442 ± 0.11	0.412 ± 0.07	0.377 ± 0.06
25	*cis*-Dihydrocarvone	1,191	0.103 ± 0.06	0.125 ± 0.02	0.131 ± 0.05	–
26	*trans*-Dihydrocarvone	1,192	–	–	–	0.124 ± 0.05
27	γ-Terpineol	1,199	1.107 ± 0.51	–	–	0.08 ± 0.002
28	Thymol	1,289	1.685 ± 0.60	1.036 ± 0.33	0.403 ± 0.37	0.526 ± 0.09
29	Carvacrol	1,298	35.88 ± 0.77	42.960 ± 3.48	38.508 ± 2.69	41.712 ± 3.25
30	Eugenol	1,356	0.042 ± 0.001	0.053 ± 0.002	0.04 ± 0.001	0.046 ± 0.006
31	Thymyl acetate	1,359	–	0.259 ± 0.009	–	–
32	α-Caryophyllene	1,408	0.125 ± 0.001	0.138 ± 0.002	0.122 ± 0.019	0.149 ± 0.007
33	*trans*-Caryophyllene	1,417	2.087 ± 0.04	2.292 ± 0.002	2.038 ± 0.25	2.411 ± 0.10
34	Bergamotene	1,418	–	0.09 ± 0.01	0.089 ± 0.003	–
35	α-Farnesene	1,440	0.101 ± 0.005	0.125 ± 0.002	–	0.082 ± 0.01
36	β-Farnesene	1,454	–	0.057 ± 0.002	–	–
37	β-Selinene	1,490	–	0.246 ± 0.02	0.245 ± 0.03	0.209 ± 0.02
38	Ledene	1,496	0.222 ± 0.10	0.28 ± 0.04	0.436 ± 0.07	0.338 ± 0.14
39	β-Bisabolene	1,505	2.502 ± 0.36	–	2.129 ± 0.04	1.869 ± 0.20
40	δ-Cadinene	1,523	0.034 ± 0.005	–	–	0.038 ± 0.01
41	Spathulenol	1,577	0.475 ± 0.03	0.605 ± 0.04	0.552 ± 0.08	1.302 ± 0.39
42	Caryophyllene oxide	1,582	0.898 ± 0.09	0.962 ± 0.06	0.804 ± 0.05	1.045 ± 0.12
Total			97.75	97.5	97.83	97.105

**Note:**

RI, Retention Index; S100, C25:S75, C50:S50, and C75:S25 refers to summer savory alone, intercropping ratio for sweet corn: summer savory (of plant densities used in respective monocultures).

Rezaei-Chiyaneh et al. (2020) demonstrated that the amounts of (E)-anethole, fenchone, and limonene in fennel were affected by intercropping. Intercropping of dill and common bean enhanced α-phellandrene and β-phellandrene content, but diminished the content of major compounds like apiole and ρ-cymene (Weisany, Raei & Ghassemi-Golezani, 2016). The improvement of EO quality of medicinal plants in the intercropping systems over monocropping might be attributed to higher resource use efficiency (Weisany, Raei & Ghassemi-Golezani, 2016; Amani Machiani et al., 2019).

### Principal component analysis (PCA) of EO composition of summer savory under different cropping patterns

**Figure 5:**
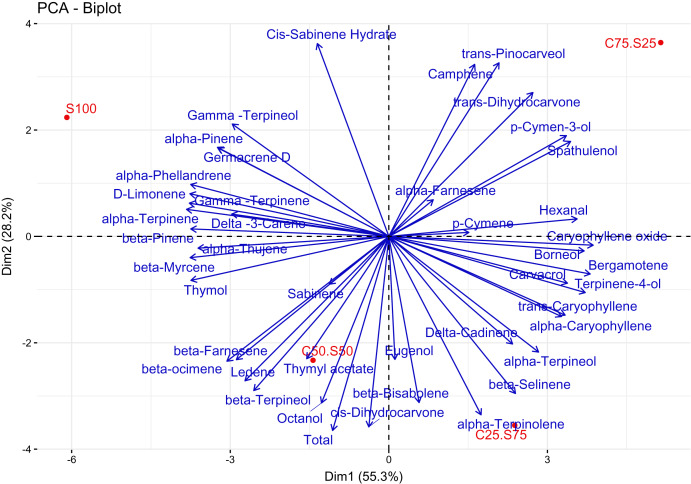
Principal component analysis (PCA) ordination biplot of EO composition of summer savory under different cropping patterns. S100, C25:S75, C50:S50, C75:S25, and C100 refer to a summer savory monoculture, intercropping ratio for sweet corn: summer savory (of plant densities used in respective monocultures), and a sweet corn monoculture.

A biplot of the first two PC demonstrated a relationship of EO composition and cropping pattern (Fig. 5). The first principal component (PC1) explained 55.3% of the total variance, while the second component (PC2) represented 28.2%. In the PCA, C75:S25 and S100 treatments were clearly discriminated based on the EO composition, but the C50:S50 and C25:S75 treatments were somewhat similar based on their EO composition (Fig. 5). Associations of major components of savory EO with cropping patterns were identified. Carvacrol associated more with C25:S75 than the other treatments, γ-terpinene and α-terpinene were highly associated with S100, and ρ-cymene was associated with C75:S25. Fallah et al. (2018) also found that cropping pattern can affect the chemical composition of dragonhead essential oil content in that intercropping soybean and dragonhead increased the content of minor components and diminished the content of major components in the EO.

## Conclusion

Sweet corn yield in monocropped fields was not significantly different from C75:S25 and C50:S50 fields. However, summer savory yield was significantly higher in summer savory alone than those in intercropped fields. Also, increasing the proportion of sweet corn caused a significant decrease in summer savory yields. Fortunately, the land equivalent ratio was >1 indicating that intercropping these two species is more efficient in terms of land and resources than growing both of these crops separately. Essential oil content in summer savory had no significant differences between S100, C50:S50, and C25:S25, however C75:S25 had the lowest EO content. Two of the major components of summer savory EO (carvacrol and ρ-cymene) were higher in C75:S25, and the other two (γ-terpinene, and α-terpinene) were higher in the summer savory monocrop. This also showed that intercropping could positively affect the quality of summer savory’s EO. These findings are important in that it will help farmers to prioritize intercropping ratios based on target crops. If farmers select sweet corn as a target species, then C75:S25 will significantly add to the yield of sweet corn; however, the yields of summer savory will be sub-optimal. Our results could help Iranian farmers select an optimal ratio of summer savory to sweet corn based on their crop targets.

## Supplemental Information

10.7717/peerj.14753/supp-1 Supplemental Information 1 Raw summer savory and sweet corn yield data.

## Additional Information and Declarations

### Data Availability

The following information was supplied regarding data availability: The raw data is available as a Supplemental File.
